# Astrogliosis and sexually dimorphic neurodegeneration and microgliosis in the olfactory bulb in Parkinson’s disease

**DOI:** 10.1038/s41531-020-00154-7

**Published:** 2021-01-21

**Authors:** Alicia Flores-Cuadrado, Daniel Saiz-Sanchez, Alicia Mohedano-Moriano, Elena Lamas-Cenjor, Victor Leon-Olmo, Alino Martinez-Marcos, Isabel Ubeda-Bañon

**Affiliations:** 1 Neuroplasticity & Neurodegeneration Laboratory, Ciudad Real Medical School, CRIB, University of Castilla-La Mancha, 13071 Ciudad Real, Spain; 2 Faculty of Health Sciences, University of Castilla-La Mancha, 45600 Talavera de la Reina, Spain

**Keywords:** Parkinson's disease, Parkinson's disease

## Abstract

Hyposmia is prodromal, and male sex is a risk marker for an enhanced likelihood ratio of Parkinson’s disease. The literature regarding olfactory bulb volume reduction is controversial, although the olfactory bulb has been largely reported as an early and preferential site for α-synucleinopathy. These pathological deposits have been correlated with neural loss in Nissl-stained material. However, microgliosis has rarely been studied, and astrogliosis has been virtually neglected. In the present report, α-synucleinopathy (α-synuclein), neurodegeneration (Neu-N), astrogliosis (GFAP), and microgliosis (Iba-1) were quantified, using specific markers and stereological methods. Disease, sex, age, disease duration, and post-mortem interval were considered variables for statistical analysis. No volumetric changes have been identified regarding disease or sex. α-Synucleinopathy was present throughout the OB, mainly concentrated on anterior olfactory nucleus. Neurodegeneration (reduction in Neu-N-positive cells) was statistically significant in the diseased group. Astrogliosis (increased GFAP labeling) and microgliosis (increased Iba-1 labeling) were significantly enhanced in the Parkinson’s disease group. When analyzed per sex, neurodegeneration and microgliosis differences are only present in men. These data constitute the demonstration of sex differences in neurodegeneration using specific neural markers, enhanced astrogliosis and increased microgliosis, also linked to male sex, in the human olfactory bulb in Parkinson’s disease.

## Introduction

Parkinson’s disease (PD) is the second most prevalent neurodegenerative disorder characterized by an associated proteinopathy (α-synucleinopathy), long prodromal period, unknown etiology^1^, and multifactorial pathogenesis^2^. According to the last *Movement Disorder Society* research criteria, masculine sex and olfactory loss are markers with a positive likelihood ratio notably increased risk factors for PD^3^. Accumulating evidence supports the neuroprotective role of estrogens in PD, given the rise of a more benign phenotype in women^4,5^. Hyposmia has also been largely reported as a prodromal deficit in PD^6^ with no apparent sexually olfactory sensory differences^7^. Interestingly, α-synuclein (α-syn) aggregates (Lewy bodies and neurites) have been described in the olfactory bulb (OB) at early neuropathological stages of the disease^8,9^. In fact, these deposits are particularly conspicuous in the OB^10–13^. A previous study in our laboratory showed that Lewy bodies and Lewy neurites were present in mitral cells and the inner plexiform layer and they were particularly abundant in the anterior olfactory nucleus. However, α-syn was scarce in olfactory cortices and it rarely co-localized with tyrosine hydroxylase^12^. It has recently been published that the α-syn are also localized in non-neuronal cells as glial and astroglial cells in the anterior olfactory nucleus^14^. Potential sexual dimorphic differences have only been occasionally addressed in the human OB^15^, showing that the number of neurons in females was 49.3% higher than males. Also, microglial cells in females proved 38.7% greater than males.

Studies using deformation-based morphometry and partial least squares have demonstrated subtle gray matter changes in regions where atrophy is related to motor and non-motor features^16^, which would be consistent with the Braak hypothesis of accumulative and predictable α-synucleinopathy in prion-like staging throughout given neural connections^17^. In the OB, literature on volumetric changes is controversial^18,19^, with studies showing significant^20–23^ or non-significant^24–26^ volume reduction in PD. A meta-analysis concluded that the volume was significantly reduced in PD patients vs. controls, and among diseased brains, the left bulb was significantly smaller^27^.

Morphometric and stereological studies using Nissl-stained post-mortem human tissue have demonstrated significant cell loss in the OB and tract, particularly in the anterior olfactory nucleus, showing correlation with disease progression and Lewy pathology^28^. No significant volumetric changes have been described, but an increase in dopaminergic cells has been described^29,30^ and it is significantly higher in males^31,32^. Increased microgliosis has also been reported in neurodegenerative proteinopathies, including PD with dementia, compared to age-matched controls^33^.

The available literature is controversial regarding volumetric changes and potential interhemispheric or sexual differences. Previous reports describing neural loss have exclusively used Nissl-stained material, and glial markers have only sporadically been used. Therefore, unbiased stereological methods and specific markers were used to rigorously measure OB volume and to quantify α-syn, the number of neurons (Fox-3, Rbfox3, or Hexaribonucleotide Binding Protein-3, Neu-N), and astroglial (glial fibrillary acidic protein, GFAP) and microglial (ionized calcium binding adaptor molecule 1, Iba-1) cells in groups of diseased and non-diseased individuals. Apart from disease, potential sexually dimorphic changes have also been considered as a variable for analysis.

## Results

### Olfactory bulb volume

**Fig. 1:**
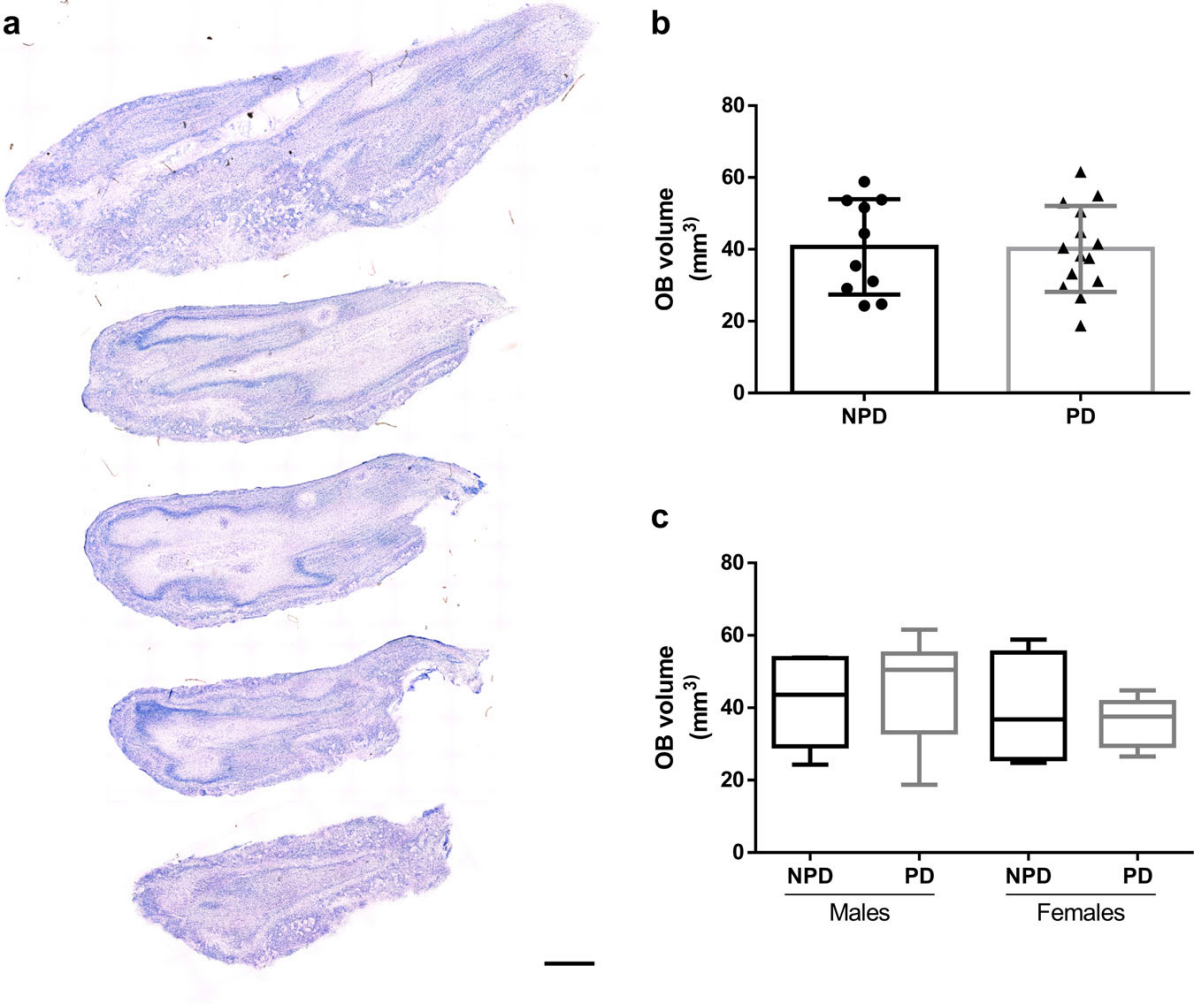
Olfactory bulb volume. Mosaic reconstruction of Nissl-stained images of horizontal sections of the human olfactory bulb. Calibration bar, 1000 µm (**a**). Graphs illustrating the volume data in Parkinson’s disease (PD) and non-Parkinson’s disease (NPD) groups (**b**) and including the sex of individuals (**c**).

Using starting sections of every OB (Fig. 1a), the total volume of the different individuals was estimated by the Cavalieri method. Data regarding estimated area, corrected volume for overprojection, coefficient of error (Gundersen, *m* = 1), section thickness, number of sections analyzed, interval between sections, grid size, number of sections, and number of elements counted are presented in Table 1. Since the data were distributed normally, an unpaired two-tailed *t*-test was applied. This did not reveal differences between PD and non-Parkinson’s disease (NPD) cases (*t*_22_ = 0.1108; *p* = 0.9128) (Fig. 1b). Two-way ANOVA did not show intersexual differences either (interaction: *F* (1, 20) = 0.3996, *p* = 0.5345; sex: *F* (1, 20) = 1.172, *p* = 0.2918; disease: *F* (1, 20) = 0.004056, *p* = 0.9499) (Fig. 1c).

**Table 1:** Estimated olfactory bulb volume.

Cases	DxAP	Estimated area (µm²)	Volume corrected for overprojection (µm³)	Coefficient of error (Gundersen), *m* = 1	Section cut thickness (µm)	Section evaluation interval	Grid size (µm)	Sections	Count
1	PD	224,250,000	53,034,400,000	0.012	50	5	250	7	3588
2	PD	187,438,000	44,768,700,000	0.012	50	5	250	6	2999
3	PD	210,875,000	50,518,800,000	0.011	50	5	250	8	3374
4	PD	229,500,000	54,968,800,000	0.007	50	5	250	7	3672
5	PD	138,375,000	33,246,900,000	0.010	50	5	250	7	2214
6	PD	131,750,000	31,187,500,000	0.015	50	5	250	5	2108
7	PD	175,000,000	41,571,900,000	0.014	50	5	250	5	2800
8	PD	157,000,000	37,537,500,000	0.010	50	5	250	6	2512
9	PD	124,312,000	29,475,000,000	0.016	50	5	250	5	1989
11	PD	160,438,000	38,250,000,000	0.015	50	5	250	5	2567
13	PD	81,500,000	18,750,000,000	0.028	50	5	250	3	1304
14	PD	170,562,000	40,415,600,000	0.014	50	5	250	5	2729
15	PD	112,812,000	26,590,600,000	0.020	50	5	250	5	1805
16	PD	257,875,000	61,578,100,000	0.013	50	5	250	6	4126
18	NPD	243,750,000	58,831,200,000	0.008	50	5	250	8	3900
19	NPD	130,688,000	31,103,100,000	0.014	50	5	250	5	2091
20	NPD	105,125,000	24,756,300,000	0.023	50	5	250	4	1682
21	NPD	223,688,000	53,815,600,000	0.013	50	5	250	8	3579
22	NPD	215,375,000	51,653,100,000	0.009	50	5	250	9	3446
23	NPD	185,438,000	44,434,400,000	0.008	50	5	250	8	2967
24	NPD	225,812,000	53,646,900,000	0.010	50	5	250	7	3613
25	NPD	123,188,000	29,128,100,000	0.013	50	5	250	5	1971
26	NPD	147,875,000	35,446,900,000	0.009	50	5	250	6	2366
27	NPD	103,375,000	24,293,800,000	0.017	50	5	250	4	1654

### α-Synucleinopathy

**Fig. 2:**
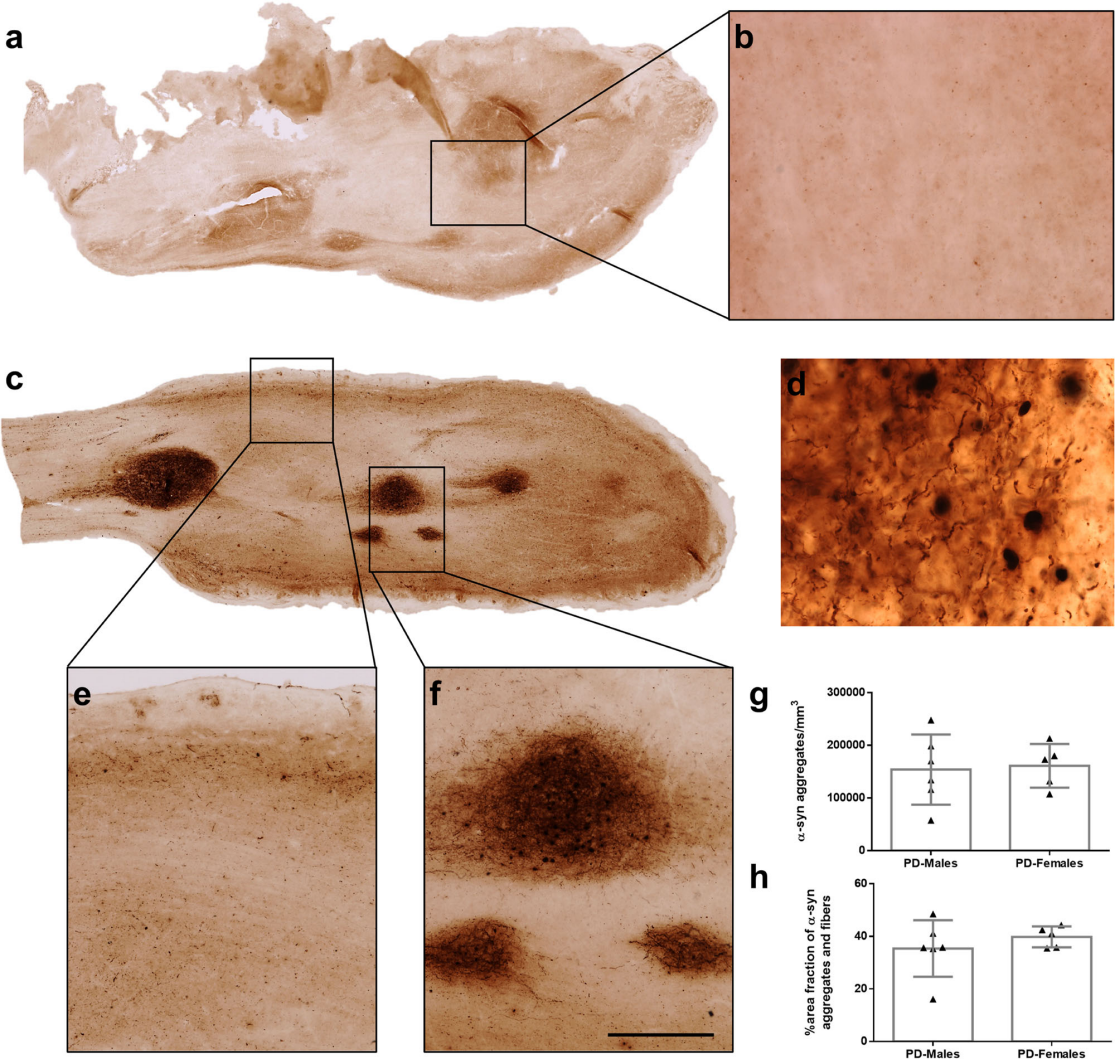
α-Synucleinopathy. Mosaic reconstruction and high-power details of α-synuclein-stained images of horizontal section of the human olfactory bulb in non-Parkinson’s disease (**a**, **b**) and Parkinson’s disease (**c**–**f**). Details of α-synuclein aggregates and fibers (**d**) in layers (**e**) and in anterior olfactory nucleus (**f**). Graphs (Mean ± SD) showing the quantification of α-synuclein’s density (aggregates/mm^3^) (**g**) and the area fraction percentage (**h**). Calibration bars, **a** and **c** 1000 µm; **b**, **e**, **f** 125 µm; **d** 50 µm.

The distribution of α-syn was stereologically analyzed by two different approaches: optical fractionator and area fraction fractionator. Data regarding elements counted, number of sections, number of sampling sites, coefficient of error (Gundersen, *m* = 1), counting frame area, sampling grade area, estimated population, volume, density, and area fraction are available in Tables 2 and 3. α-Syn aggregates and fibers were not observed in NPD (Fig. 2a, b), whereas those were present throughout the OB, mainly concentrated on anterior olfactory nucleus (dark staining) of the 11 PD cases used in this study (Fig. 2c–f). Density, which was obtained after dividing the estimated population (optical fractionator probe) by the volume, was similar in both males and females, being 154,100 ± 66,617 and 161,151 ± 41,570 α-syn aggregates/mm^3^, respectively (unpaired two-tailed *t*-test, *t*_9_ = 0.2048, *p* = 0.8423) (Fig. 2g). The area fraction of α-syn (area fraction fractionator probe), which measured the percentage of area occupied by aggregates and fibers, was also analogous between males (35.38 ± 10.74%) and females (39.80 ± 3.978%) (Mann–Whitney *U* = 11, *p* = 0.5281) (Fig. 2h).

**Table 2:** Estimated olfactory bulb α-synuclein number and density.

Cases	DxAP	Total markers counted	Number of sections	Number of sampling sites	Coefficient of error (Gundersen), *m* = 1	Counting frame area (XY) (µm²)	Sampling grid area (XY) (µm²)	Estimated population using mean section thickness	Measured volume (mm³)	Density (cell/mm^3^)
5	PD	499	6	236	0.05	900	250,000	829,533.44	14.39	57,666.56
7	PD	481	4	70	0.05	900	1,000,000	3,193,458.75	17.73	180,129.32
8	PD	499	5	62	0.05	900	1,000,000	3,342,852.25	15.70	212,874.43
9	PD	413	5	61	0.05	900	1,000,000	2,718,442.00	15.70	173,182.26
10	PD	1744	3	196	0.03	900	250,000	2,938,421.25	11.86	247,775.67
11	PD	395	4	77	0.06	900	1,000,000	2,598,875.75	19.65	132,230.04
12	PD	203	7	51	0.07	900	1,000,000	1,333,214.38	11.52	115,769.61
14	PD	460	6	93	0.05	900	1,000,000	3,032,370.50	22.55	134,480.95
15	PD	296	5	75	0.06	900	1,000,000	1,948,283.75	18.15	107,339.32
16	PD	508	3	73	0.07	900	250,000	854,206.94	4.29	198,906.27
17	PD	648	3	108	0.05	900	250,000	1,088,870.25	6.40	170,003.69

**Table 3:** Estimated olfactory bulb α-synuclein area fraction.

Cases	DxAP	Marker count	Total markers counted	Number of sections	Number of sampling sites	Grid spacing (µm)	Coefficient of error (Gundersen), *m* = 1	Counting frame area (XY) (µm²)	Sampling grid area (XY) (µm²)	Area fraction (%)
5	PD	465	2891	6	79	15	0.038	10,000	250,000	16.08
7	PD	1331	3007	4	78	15	0.035	10,000	1,000,000	44.26
8	PD	1163	2740	5	67	15	0.021	10,000	1,000,000	42.45
9	PD	996	2807	5	72	15	0.019	10,000	1,000,000	35.48
10	PD	727	2031	3	51	15	0.031	10,000	250,000	35.80
11	PD	1407	3430	4	86	15	0.035	10,000	1,000,000	41.02
12	PD	934	1926	7	51	15	0.025	10,000	1,000,000	48.49
14	PD	1258	3064	6	95	15	0.026	10,000	1,000,000	41.06
15	PD	1326	3707	5	92	15	0.040	10,000	1,000,000	35.77
16	PD	254	721	3	18	15	0.041	10,000	250,000	35.23
17	PD	424	1190	3	32	15	0.027	10,000	250,000	35.63

### Neurodegeneration

**Fig. 3:**
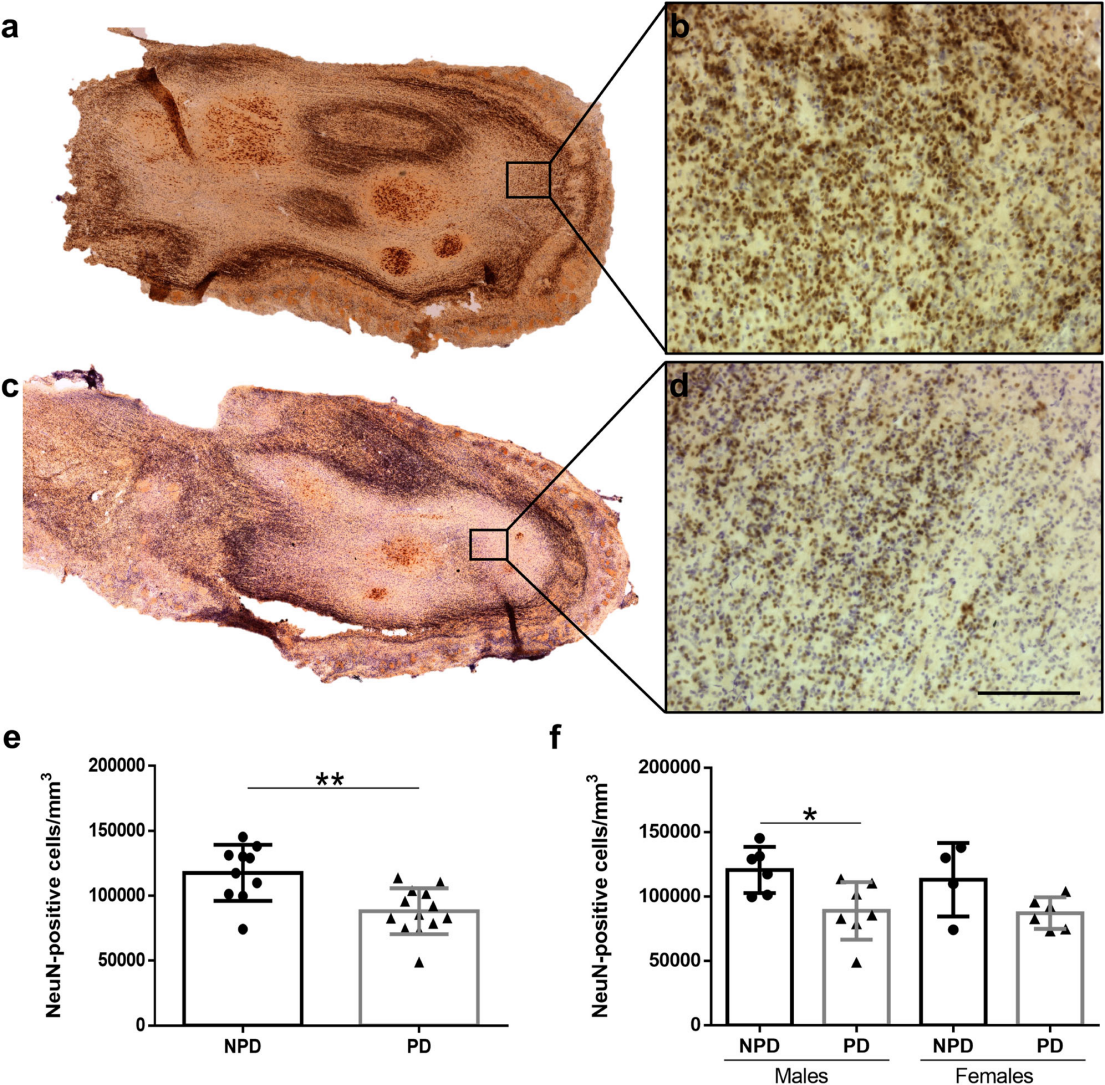
Neurodegeneration. Mosaic reconstruction of Neu-N-immunoreacted images of horizontal sections of the human olfactory bulb in non-Parkinson’s disease (**a**) and a Parkinson’s disease case (**c**) and the corresponding high-power details (**b**, **d**). Calibration bars, **a**, **c** 1000 µm; **b**, **d** 100 µm. Graphs (Mean ± SD) illustrating the density of Neu-N-positive cells in the Parkinson’s disease (PD) and non-Parkinson’s disease (NPD) groups (**e**) and including the sex of individuals (**f**).

Neu-N labeling in the NPD (Fig. 3a, b) and PD (Fig. 3c, d) groups was analyzed using an optical fractionator probe. Data regarding elements counted, number of sections, number of sampling sites, coefficient of error (Gundersen, *m* = 1), counting frame area, sampling grade area, estimated population, volume, and density are available in Table 4. Statistical analysis revealed a significant neural loss in PD compared to NPD cases (unpaired two-tailed *t*-test, *t*_21_ = 3.617, *p* = 0.0016) (Fig. 3e). Two-way ANOVA including neurodegeneration and sex (between and within) demonstrated that degeneration was only significant in males (interaction: *F* (1, 19) = 0.1145, *p* = 0.7388; sex: *F* (1, 19) = 0.2889, *p* = 0.5965; disease: *F* (1, 19) = 11.21, *p* = 0.0034). Given that the disease factor was significant, to identify this difference, the analysis of each sex was separately carried out, which showed differences only in males (male: unpaired two-tailed *t*-test, *t*_11_ = 2.794, *p* = 0.0175; female: unpaired two-tailed *t*-test, *t*_8_ = 2.017, *p* = 0.0784) (Fig. 3f).

**Table 4:** Estimated olfactory bulb neuron number and density.

Cases	DxAP	Total markers counted	Number of sections	Number of sampling sites	Coefficient of error (Gundersen), *m* = 1	Counting frame area (XY) (µm²)	Sampling grid area (XY) (µm²)	Estimated population using mean section thickness	Measured volume (mm^3^)	Density (cell/mm^3^)
1	PD	493	5	114	0.05	900	1,000,000	3,325,011.11	29.23	113,724.97
2	PD	334	4	111	0.06	900	640,000	1,436,942.22	17.36	82,790.34
3	PD	477	5	132	0.05	900	1,000,000	3,263,984.62	32.05	101,832.13
4	PD	394	5	131	0.05	900	1,000,000	2,756,540.74	32.21	85,565.67
5	PD	944	2	159	0.06	900	90,000	584,336	3.45	169,378.65
7	PD	226	5	84	0.08	900	1,000,000	1,582,000	21.15	74,796.23
8	PD	212	5	61	0.07	900	1,000,000	1,457,303.70	15.79	92,261.27
9	PD	246	5	67	0.07	900	1,000,000	1,648,200	17.21	95,743.75
10	PD	166	3	49	0.09	900	640,000	714,168.89	8.61	82,946.54
11	PD	473	4	132	0.07	900	640,000	2,085,404.44	20.06	103,930.89
13	PD	330	2	165	0.10	900	90,000	204,380	4.18	48,931.73
14	PD	477	4	122	0.05	900	640,000	2,071,381.33	18.77	110,347.14
15	PD	174	5	69	0.08	900	1,000,000	1,178,044.44	16.07	73,324.40
16	PD	380	4	140	0.06	900	640,000	1,700,598.52	21.60	78,716.11
18	NPD	292	3	67	0.07	900	640,000	1,306,083.56	10.04	130,098.37
19	NPD	269	5	51	0.07	900	1,000,000	1,877,022.22	12.93	145,193.83
20	NPD	134	3	54	0.09	900	640,000	598,731.85	8.06	74,252.84
21	NPD	405	5	101	0.05	900	1,000,000	2,845,500	24.23	117,437.06
22	NPD	553	7	120	0.05	900	1,000,000	3,813,651.85	29.07	131,207.08
23	NPD	250	3	60	0.07	900	640,000	1,110,518.52	10.10	109,899.01
24	NPD	499	6	107	0.05	900	1,000,000	3,509,633.33	27.18	129,132.71
25	NPD	194	5	41	0.08	900	1,000,000	1,354,407.41	9.80	138,138.73
26	NPD	421	3	123	0.07	900	90,000	258,353.67	2.55	101,168.37
28	NPD	358	6	106	0.07	900	1,000,000	2,507,325.93	25.12	99,803.60

To confirm whether neurodegeneration was correlated to α-synucleinopathy, linear regression and Pearson test were carried out. The results did not show that the density of Neu-N (cells/mm^3^) was decreased as density of α-syn (aggregates/mm^3^) increased (Pearson *r* = −0.2418, *p* = 0.5639) (Supplementary Fig. 1a).

### Astrogliosis

**Fig. 4:**
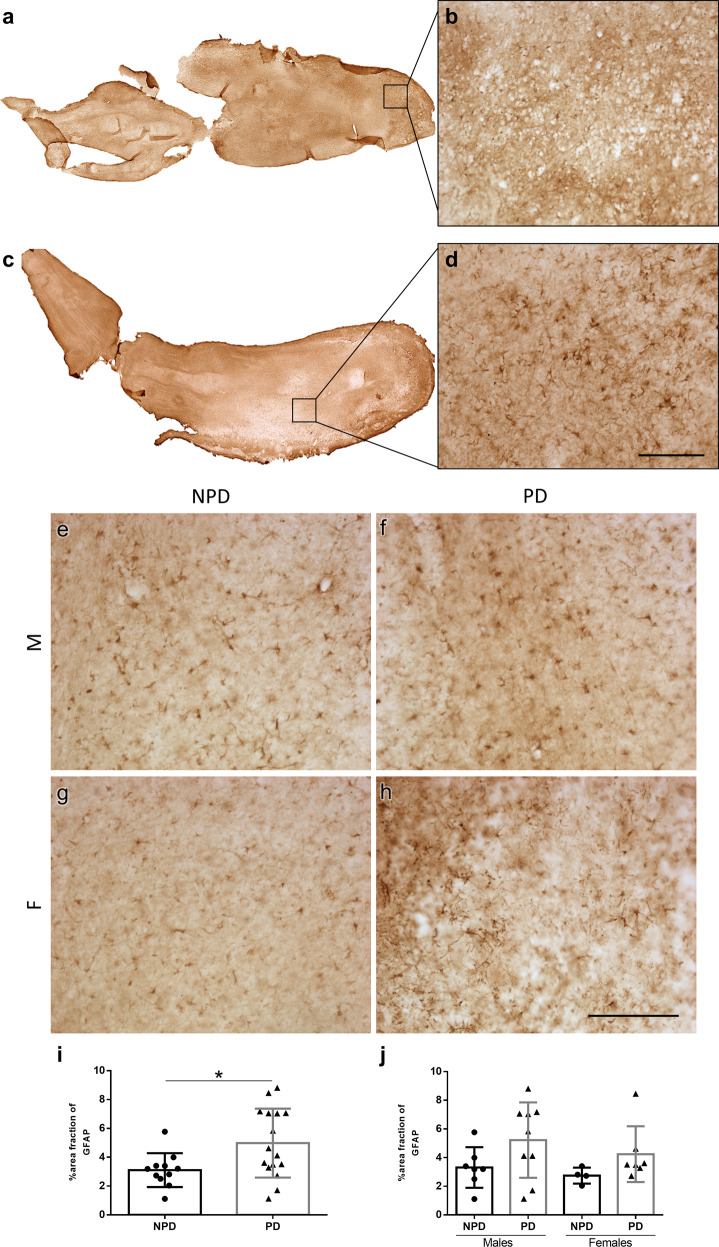
Astrogliosis. Mosaic reconstruction of GFAP-immunolabeled images of horizontal sections of the human olfactory bulb in non-Parkinson’s disease (**a**) and a Parkinson’s disease case (**c**) and the corresponding high-magnification (**b**, **d**). Images of GFAP-immunoreacted horizontal sections of the human olfactory bulb in non-Parkinson’s disease (**e**, **g**) and a Parkinson’s disease case (**f**, **h**) in both sexes. M: males (**e**, **f**); F: females (**g**, **h**). Calibration bars, **a**, **c** 1000 µm; **b**–**h** 125 µm. Graphs (Mean ± SD) represent the percentage of the area fraction of GFAP in the Parkinson’s disease (PD) and non-Parkinson’s disease (NPD) groups (**i**) and including the sex of individuals (**j**).

Analysis of GFAP labeling in both sexes and in NPD (Fig. 4a, b, e, g) and PD cases (Fig. 4c, d, f, h) using the area fraction method demonstrated a significant increase in diseased compared to non-diseased brains (unpaired two-tailed *t*-test, *t*_25_ = 2.394, *p* = 0.0245) (Fig. 4i). When analyzed per sex, a non-significant trend toward astrogliosis in PD was observed (two-way ANOVA: interaction: *F* (1, 23) = 0.06800, *p* = 0.7966; sex: *F* (1, 23) = 0.9359, *p* = 0.3434; disease: *F* (1, 23) = 4.545, *p* = 0.0439). Given that the disease factor was significant, to identify this difference, the analysis of each sex was separately carried out, which did not show differences in either sex (male: unpaired two-tailed *t*-test, *t*_14_ = 1.731, *p* = 0.1054; female: unpaired two-tailed *t*-test, *t*_9_ = 1.474, *p* = 0.1745) (Fig. 4j). The correlation of α-syn with GFAP was analyzed by linear regression and Pearson test using area fraction values (Pearson *r* = −0.4484, *p* = 0.1937). No correlation was detected (Supplementary Fig. 1b).

### Microgliosis

**Fig. 5:**
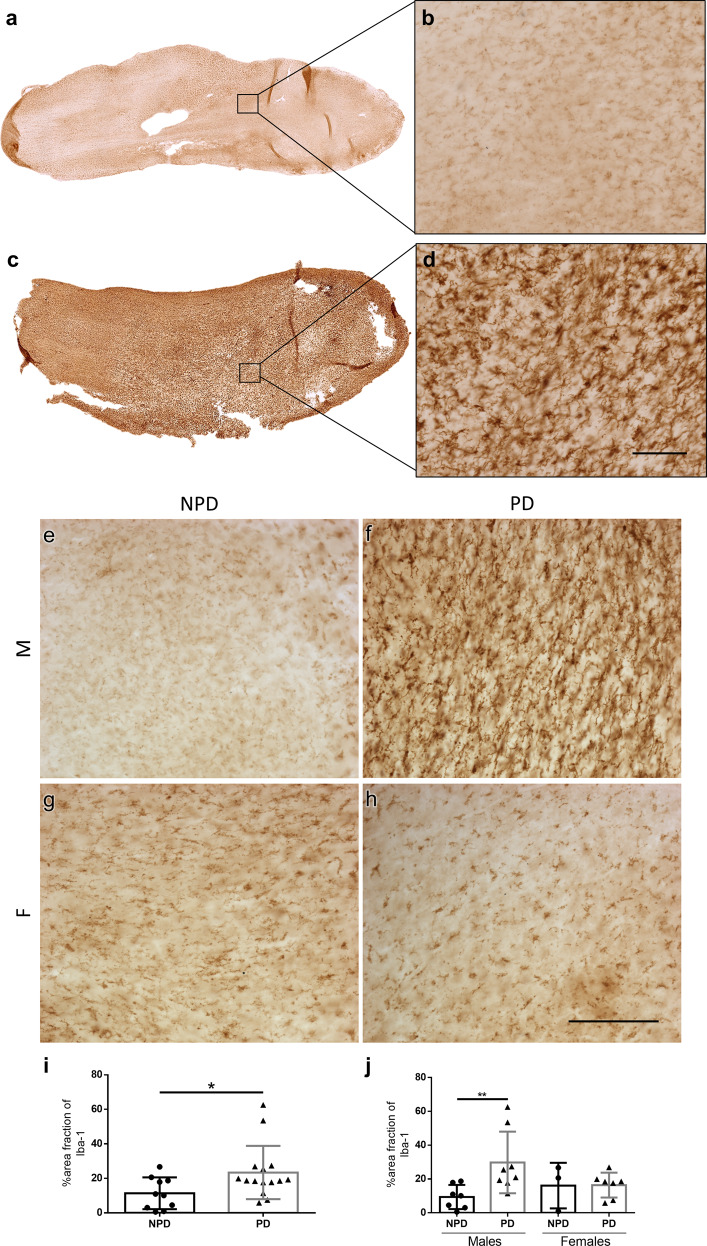
Microgliosis. Mosaic reconstruction of Iba-1-immunolabeled images of horizontal sections of the human olfactory bulb in non-Parkinson’s disease (**a**) and a Parkinson’s disease case (**c**) and the corresponding high-magnification (**b**, **d**). Images of Iba-1 immunoreactive horizontal sections of the human olfactory bulb in non-Parkinson’s disease (**e**, **g**) and a Parkinson’s disease case (**f**, **h**) in both sexes. M: males (**e**, **f**); F: females (**g**, **h**). Calibration bars, **a**, **c** 1000 µm; **b**–**h** 125 µm. Graphs (Mean ± SD) represent the percentage of the area fraction of Iba-1 in the Parkinson’s disease (PD) and non-Parkinson’s disease (NPD) groups (**i**) and including the sex of individuals (**j**).

Iba-1 labeling in NPD (Fig. 5a, b, e, g) and PD cases of both sexes was analyzed (Fig. 5c, d, f, h). Area fraction quantification showed a significant increase in the percentage of PD patients compared to NPD patients (Mann–Whitney *U* = 37.50, *p* = 0.0370) (Fig. 5i). Analysis per sex showed that this difference was even greater in males, but it does not exist in females (two-way ANOVA: interaction: *F* (1, 21) = 3.530, *p* = 0.0742; sex: *F* (1, 21) = 0.3932, *p* = 0.5374; disease: *F* (1, 21) = 3.759, *p* = 0.0661) (Fig. 5j). Regarding to the involvement the α-syn with microgliosis, linear regression and Pearson test were carried out. The area fraction of Iba-1 was not correlated to area fraction of α-syn (Pearson *r* = −0.2275, *p* = 0.5560) (Supplementary Fig. 1c).

### Further correlations

Apart from age and sex, post-mortem human OBs are highly heterogeneous due to other factors such as interindividual differences, disease duration, and post-mortem interval. To analyze this variability, all markers (density of α-syn, density of Neu-N, area fraction of Iba-1, and area fraction of GFAP) were compared to disease duration and post-mortem interval. No significant correlations were found (Supplementary Figs. 2 and 3).

## Discussion

**Fig. 6:**
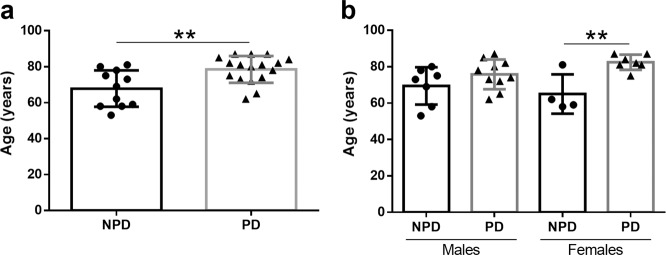
Age and sex. Graphs illustrating age (**a**) and age and sex (**b**) in Parkinson’s disease (PD) and non-Parkinson’s disease (NPD) groups.

Demographically, the data presented in this report demonstrate that the group of PD-diagnosed individuals was significantly older than the NPD group. However, when estimated per sex, this age difference was only detected in the female group (Fig. 6). The data presented in this report demonstrate no volumetric changes between PD and NPD cases, either between sex (Fig. 1). α-syn quantification did not show differences between males and females, and the labeling was concentrated on anterior olfactory nucleus (Fig. 2). Neurodegeneration, considered a reduction in Neu-N-positive cells, was demonstrated in the diseased group and was also significant in men (Fig. 3). However, astrogliosis, demonstrated by the increased area fraction of GFAP labeling, was significant in the PD group compared with the NPD group, but this difference was not significant when analyzed per sex (Fig. 4). Finally, microgliosis, assumed by augmented labeling of Iba-1, was significantly found in the PD group. When studied per sex, this difference was only found in men (Fig. 5). Since the PD and NPD groups of men were age matched, this microgliosis can be attributed to a sex effect. To the best of our knowledge, microgliosis linked to masculine sex was previously unreported.

There are sex differences in the symptomatology, pathophysiology, susceptibility, medication use, levodopa bioavailability, among others in PD^34,35^. Focusing on symptomatology, pathophysiology, and susceptibility, these differences could be due to exogenous and endogenous estrogens^34,36^. As a result, the incidence in men is greater than women (approximately 3:2 ratio)^37^. In fact, most women develop PD after menopause, confirmed by a positive correlation between age of PD onset and duration of fertile life^34,35^. Despite promising results about the estrogen neuroprotective effects in neurotoxic animal models^36,38–40^, these might be not corroborated in the human disease. According to literature, a number of clinical studies had been performed to characterize the correlation between estrogens exposure and PD risk; however, the role of estrogens remains controversial due to disparity findings^34,36^. Estrogens have been associated to different roles: mediators in oxidative stress, mitochondrial dysfunction, and protecting the nigrostriatal dopaminergic pathway^34^. The role of estrogens in this pathway has been studied using both gonadally intact and ovariectomized animals, showing that estrogens increase the nigral dopaminergic synthesis and the release of dopamine from nigral axon terminals within the striatum^36^. In humans, estrogens could affect clinical symptomatology, to improve^41^ or worsen^42^ motor symptoms. Therefore, it has been suggested other several variables as age, estrogen dose and formula, and timing and length of doses period^36^. In addition, it has been described other factors, as dysmorphological changes in the basal ganglia, younger women have a higher baseline number of dopaminergic cells in substantia nigra and also, sex chromosomes could be involved in the dopamine system.

As mentioned above, symptomatology is affected by sex. Regarding motor symptoms, women present tremor as initial symptoms of PD and worse UPDRS instability score in comparison with men^35^. According to non-motor symptoms, women have higher prevalence and severity of the sleep/fatigue, mood/apathy, and pain symptoms, whereas men have more urinary symptoms. Meanwhile, hyposmia, which has also been largely reported as a prodromal deficit in PD^6^, does not show apparent sexually olfactory sensory differences^7^.

Previous reports on OB volumetric changes in PD have been controversial^18^. Some magnetic resonance studies report volume reduction with the disease^20–23^, whereas others do not observe changes^24–26^. A recent meta-analysis concludes a volume reduction in PD, and among them, further significant reduction in the left OB^27^. Our present data using stereology match previous observations^30^ and do not report significant volume reduction regarding disease and sex (Fig. 1).

α-Synucleinopathy was localized throughout the OB, mainly converging on the different subdivisions of the anterior olfactory nucleus of the 11 PD cases used in this study, as previously described different reports^8,12,14^. The explanation that the densest labeling in the olfactory system was present in the anterior olfactory nucleus cannot be exclusively clarified from OB afferent connections, but it might be due to its multiple centripetal, centrifugal, commissural, associational, and non-olfactory connections^43^. Although our results of α-syn did not show differences between males and females (Fig. 2), it has been reported that once again the estrogens may be protective in PD preventing Lewy body formation, destabilizing the fibrillization and the aggregation of α-syn^44^.

Regarding neurodegeneration, early studies have reported neuronal loss (using Nissl-stained material) in the anterior olfactory nucleus^28^ correlating with pathology. Our current data, using specific neural markers, in agreement with the previous literature, also describe that this occurs in men (Fig. 3). However, the positive correlation was not found between the density of α-syn and the density of Neu-N, that means, the density of Neu-N (cells/mm^3^) was significantly decreased as density of α-syn (aggregates/mm^3^) increased (Supplementary Fig. 1a).

Astrocytes are critical for brain function, including homeostasis, neuronal metabolism, and blood–brain barrier maintenance, and have been involved in the inflammatory response (astrogliosis) associated with PD^45^. α-Syn is predominantly expressed in neurons, but it also aggregates in astrocytes, disrupting their function^46^. Early reports on astroglial marker (GFAP) using western blot with human brain homogenates indicated GFAP low levels in the substantia nigra correlated inversely with α-syn accumulation in patients with PD^47^. To the best of our knowledge, our current data are a rigorous stereological quantification of astrogliosis in the human OB in PD (Fig. 4). Our results could be correlated to a recent report by Stevenson and colleagues^14^ describing intracellular α-syn inclusions in astrocytes.

Microglia, under physiological conditions, display immune and phagocytic capacity, whereas they are activated in pathological circumstances such as neurodegeneration and can migrate, engulf debris, sense pathological markers, and secrete pro- and anti-inflammatory compounds^48,49^. The use of the term “inflammation” to describe the complex and heterogeneous interactions carried out by microglia in PD is too simplistic, and perhaps “immune dysregulation” would be a better definition^50^. The potential role of microglia in disease progression, particularly the interaction with dopaminergic neurons, may explain the specificity of the pathology in given brain regions such as the substantia nigra. Dopaminergic neural signals to microglia include α-syn and substance P^51^, both of which, apart from dopaminergic neurons, are particularly abundant in the OB^12^. In fact, increased microgliosis has been reported in the human OB of demented PD patients^33^, which is in agreement with our findings (Fig. 5). In addition, microglia contain intracellular α-syn inclusion (7.78%), as occurs in astrocytes^14^. Regarding the sex effect of microgliosis, it has been proposed in neurodegeneration models that inflammation endangers neuronal survival, but estrogens reduce microglial inflammation, which could protect neurons in female brains^34,52,53^. This would help to explain our current results show the sex dimorphic microglial response in the human brain in PD (Fig. 5).

Collectively, the current data suggest that in regions that are involved in α-synucleinopathy early and preferentially, such as the OB, neurodegeneration, and microgliosis (linked to masculine sex) and astrogliosis occur simultaneously. Evidence suggests that activated glial cells facilitate clearance of pathological proteins, but recent studies suggest that astroglia and microglia promote spreading of proteinopathies and facilitate disease progression^54^. Therefore, the OB constitutes a fundamental “hub” from this connectomic perspective in which proteomic analysis would be essential to identify biomarker proteins^55–57^.

## Methods

### Human samples

In the present study, tissue from *N* = 28 individuals (with or without PD diagnosis; PD = 17 and NPD = 11, respectively) was used (Table 5). The average brain weight of PD cases (1224 ± 127.6 g) was not significantly different from that of the NPD group (1172 ± 158.5 g) (unpaired two-tailed *t*-test, *t*_26_ = 0.9470, *p* = 0.3524). Samples and data from donors included in this study were provided by the IDIBAPS, BIOBANC-MUR, BTCIEN, and BPA, integrated in the Spanish National Biobanks Network, and they were processed following standard operating procedures with the appropriate approval of the Ethical and Scientific Committees. These biobanks protocols involved written informed consent of donors. All experiments carried out in this work were authorized by the Ethical Committee of Clinical Research of the Ciudad Real University Hospital (SAF2016-75768-R).

**Table 5:** Demographic and clinicopathological features of the individuals with or without Parkinson’s disease diagnosis.

Cases	DxAP	Sex	Age (years)	PMD (hh:mm)	Brain weight (g)	Stage	Disease duration (years)	Original fixation	Cause of death	Iba-1	Neu-N	Cavalieri	GFAP	α-Syn
1	PD	M	73	1:00	1450	Braak 6	1,5	Fd	Cardiorespiratory arrest	X	X	X	X	
2	PD	F	82	2:00	1300	Braak 5	22	Fd	Cardiorespiratory arrest (immediate), intestinal ischemia (secondary)	X	X	X	X	
3	PD	M	82	5:00	m.d.	Braak 6	8	Fd	m.d.	X	X	X	X	
4	PD	M	65	m.d.	1305	Braak 6	9	Fd	m.d.	X	X	X	X	
5	PD	M	80	m.d.	1231	Braak 5	m.d.	Fd	m.d.	X	X	X	X	X
6	PD	F	87	2:00	1100	Braak 6	10	Fr	Cardiorespiratory arrest	X		X	X	
7	PD	F	81	12:20	1105	Braak 6	13	Fr	Urinary sepsis	X	X	X	X	X
8	PD	F	84	4:30	1050	Braak 4	22	Fr	Acute myocardial infarction	X	X	X	X	X
9	PD	F	81	6:30	1010	Braak 4	12	Fr	Hepatic carcinoma	X	X	X	X	X
10	PD	M	85	12:15	1355	Braak 5	12	Fr	Bronchoaspiration	X	X		X	X
11	PD	F	87	7:00	1140	Braak 6	20	Fr	Bronchoaspiration	X	X	X	X	X
12	PD	M	74	8:00	1305	Braak 5	19	Fr	Pneumonia	X			X	X
13	PD	M	87	15:15	m.d.	Braak 5	m.d.	Fr	m.d.	X	X	X	X	
14	PD	M	78	5:15	1210	Braak 5	m.d.	Fr	Bronchoaspiration	X	X	X	X	X
15	PD	F	75	3:45	m.d.	Braak 5	m.d.	Fr	m.d.	X	X	X	X	X
16	PD	M	72	6:00	1160	Braak 6	15	Fr	Advanced cognitive impairment + renal insufficiency	X	X	X	X	X
17	PD	M	62	13:30	1355	Braak 5	12	Fr	Cardiorespiratory arrest					X
18	NPD	F	62	2:00	1050	–	–	Fr	Cardiorespiratory arrest (immediate), Multiple organ dysfunction syndrome (secondary)	X	X	X	X	
19	NPD	M	58	6:00	1500	–	–	Fr	Acute myocardial infarction (immediate), ischemic heart disease (secondary)	X	X	X	X	
20	NPD	F	59	2:00	1200	–	–	Fr	Severe acute pancreatitis (immediate), Cardiorespiratory arrest (secondary)	X	X	X	X	
21	NPD	M	53	5:00	1300	–	–	Fr	Cardiorespiratory arrest (immediate), rectal carcinoma (secondary)	X	X	X	X	
22	NPD	M	78	4:00	1100	–	–	Fr	Respiratory insufficiency (immediate), lung cancer (secondary)	X	X	X	X	
23	NPD	F	81	5:00	1100	–	–	Fd	Multiple organ dysfunction syndrome	X	X	X	X	
24	NPD	M	75	4:00	1250	–	–	Fd	Multiple organ dysfunction syndrome	X	X	X	X	
25	NPD	F	58		944	–	–	Fd		X	X	X	X	
26	NPD	M	80	10:00	1310	–	–	Fr	Piriformis sinus carcinoma	X	X	X	X	
27	NPD	M	69	10:15	1110	–	–	Fr	Pneumonia	X		X	X	
28	NPD	M	73	6:10	1030	–	–	Fr	Bronchoaspiration	X	X		X	

*DxAP* neuropathological diagnosis, *PMD* post-mortem duration, *PD* Parkinson’s disease, *NPD* non-Parkinson’s disease, *M* male, *F* female, *m.d.* missing data, *Fd* formaldehyde, *Fr* frozen no-fix.

### Age and sex

Since aging could be potentially relevant in subsequent analyses, ages between the NPD (mean 67.82 ± 10.15 years) and PD (mean 78.73 ± 7.45 years) groups were compared. Data of individuals were normally distributed, and the unpaired two-tailed *t*-test revealed a significantly older age for PD compared to NPD cases (*t*_26_ = 3.223; *p* = 0.0034) (Fig. 6a). Two-way ANOVA including age and sex (between and within) demonstrated that only women with PD (mean 82.43 ± 4.16) years) were significantly older than NPD females (mean 65 ± 10.80 years) [interaction: *F* (1, 24) = 2.739, *p* = 0.1110; sex: *F* (1,24) = 0.1084, *p* = 0.7449; disease: *F* (1, 24) = 12.68, *p* = 0.0016) (Fig. 6b)].

### Histologic procedures

Tissues were received either frozen or immersed in formaldehyde (Table 5). As previously described in our laboratory^12^, to standardize the conditions of the samples received, all of them were immersed in fresh phosphate-buffered 4% paraformaldehyde for 45 days. Afterward, bulbs were kept in a phosphate-buffered solution of 2% dimethyl sulfoxide (DMSO) for 48 h and 10% glycerol and for 48 h in a phosphate-buffered solution of 2% DMSO and 20% glycerol for cryoprotection. It is important to note that the fixation and thawing processes may have affected the thickness of the samples, but this effect was stereologically standardized thereafter for all cases (see below). Using a freezing sliding microtome, horizontal sections (50 µm) were obtained. Five series of sections were collected; one was mounted and counterstained with toluidine blue, and the remaining sections were kept in a phosphate-buffered solution of 20% glycerol and 30% ethylene glycol at −20 °C for future processing: α-syn, Neu-N, Iba-1, GFAP immunohistochemistry’s.

### Immunohistochemistry

Immunohistochemistry protocol was provided by neuropathologists from Spanish Biobanks and it is routinely used in our laboratory. Tissue was boiled under pressure for 2 min and 30 s in citrate buffer and after that, sections were immersed in formic acid for 3 min and rinsed in phosphate-buffered saline (PBS, pH 7.4) to unmask antigenicity. This double unmasking procedure is used since samples were kept in paraformaldehyde during long periods of time at Biobanks. Endogenous peroxidase activity was inhibited by a 30-min bath in 1% H_2_O_2_ in PBS. Sections were incubated overnight in primary antibody (Table 6), subsequently incubated for 2 h in secondary antibody (biotinylated horse anti-mouse or anti-rabbit IgG (H+L) Vector Laboratories, 1:200 in blocking buffer) and finally incubated in avidin–biotin complex (ABC standard, Vector, containing 0.3% TX-100) and reacted using 0.025% 3,3ʹ-diaminobenzidine and 0.1% H_2_O_2_. Sections were mounted, dried, dehydrated, and coverslipped with DPX (Sigma-Aldrich).

**Table 6:** Antibodies used in the present study.

Antigen	Manufacturer	Cat no.	Species	Dilution	BB	Incubation
Iba-1	Wako	019–19741	Rabbit polyclonal antibody	1:2000	PBS + 0.1% TX-100	4 °C overnight
GFAP	DAKO	Z0334	Rabbit polyclonal antibody	1:10,000	PBS + 0.1% TX-100 + 10% Normal horse serum	4 °C overnight
Neu-n	Abcam	Ab104225	Rabbit polyclonal antibody	1:500	PBS + 0.3% TX-100	Room temperature overnight
α-Syn	Novocastra^TM^ Leica Biosystems	NCL-L- α-syn	Mouse monoclonal antibody	1:20	PBS + 0.3% TX-100	4 °C 48 h

The antibody NCL-L-α-syn (previously called KM51) has been widely used for Parkinson’s disease post-mortem diagnosis at Biobanks^58,59^ and also was reported as one of the best antibodies against human α-syn^60,61^.

### Stereological quantification

Stereological quantification was performed using Stereo Investigator software (MBF Bioscience coupled to a Zeiss Axio Imager M2 microscope). The OB volume was calculated using the Cavalieri estimator. Boundaries of sections of the OB, excluding the olfactory peduncle, were outlined using a low magnification objective (Plan-Neofluar 1x/0.025, Ref. 420300-9900-000) (Table 1). Two different stereological approaches were carried out to quantify α-syn: optical fractionator (estimation of the total number of aggregates) and area fraction fractionator probes (percentage of area fraction occupied by aggregates and fibers) (Tables 2 and 3). Optical fractionator is an unbiased probe which is not influenced by the size, shape, spatial orientation, or spatial distribution of the cells under study. The first step was to draw the boundaries of 11 PD cases (*n* = 51) and a total 1102 sites were analyzed. The parameters used were 30 × 30 µm counting frame size, 2 µm guard zone, 18 µm height dissector, and 500 × 500 or 1000 × 1000 µm of sampling grid size depending on the sections analyzed per case (3 or 4–6, respectively). The counting frame is composed of red lines (rejection lines) and green lines (acceptance region). Therefore, the counting rule was that α-syn aggregate was counted if it lies entirely within the counting frame or if it touches the green line without touching the red line. To clarify how the height dissector is obtained, several random measurements of the thickness of each section are taken, and an average is established. This value is maintained throughout the quantification and it is calculated for each case. The guard zones prevent possible artifacts that the tissue may have suffered in the upper and lower surfaces during fixation, cutting, or immunohistochemistry. These guard zones reduce the available section thickness that can be used for counting.

Area fraction fractionator was also used due to the heterogeneity of α-syn aggregates size and fibers. This test allows to estimate percentage of area of α-syn also in neuropile. A random two-dimensional virtual square was superimposed on the region; and an array of points was in each sampling box. One marker is used on points for one type of staining (α-syn), while another marker is used for the rest of the tissue (neuropile). The number of points over α-syn was divided by the total number of points (reference) to get an estimate of the area.

The density of Neu-N-positive cells was calculated using an optical fractionator probe (Plan Apochromat, 63x/1.4, oil lens, Ref. 420782–9900). Boundaries of PD (*n* = 58) and NPD (*n* = 46) cases were traced, and a total of 2366 sites were analyzed. The parameters used were 30 × 30 µm counting frame size, 1 µm guard zone, 15 µm height dissector, and 300 × 300, 800 × 800, or 1000 × 1000 µm of sampling grid size depending on the sections analyzed per case (2, 3–4, or 5–6, respectively) (Table 4).

To quantify the intricate labeling of Iba-1 and GFAP markers and based on the steps of stereological area fraction fractionator method described above, an Image J analysis of the area fraction method was performed. This probe included random region-of-interest selection, photography, and ImageJ analysis. Following an unbiased protocol, first, a millimetric transparent grid was randomly overlapped in the slide and crossed matched to the tissue identified simulating the previously mentioned array of points (Supplementary Fig. 4a); second, to maintain randomness for image capture, odd and even numbers were randomly assigned to each human case (Supplementary Fig. 4b). In odd-numbered cases, the images were taken from the first cross-matching of the tissue following this cross-matching sequence for the first line: 3-5-7 (Supplementary Fig. 4c). In even-numbered cases, the images were captured in the second cross-matching following this sequence: 4-6-8 (Supplementary Fig. 4d). Images were captured (Iba-1, *n* = 887 and GFAP, *n* = 554) using a Nikon Eclipse 80i microscope (Plan Apo 20x/0.75, Ref. MRD00205). Third, images were processed with an ImageJ protocol^62^ further implemented in a macro. Briefly, images were converted to 8-bit grayscale, and the histogram was obtained. The histogram mode is the most representative value of the image, in all cases it is the neuropile (background). Then, the histogram mode was multiplied by 0.6 (Iba-1 labeling) or 0.7–0.85 (GFAP labeling) to obtain the threshold for considering specific cell labeling from background or Nissl-counterstain and to measure the area fraction. The area fraction is an average percent of each picture labeled for each case.

### Statistical analysis

Statistical analysis was carried out using GraphPad Prism® software (v6.01; La Jolla, CA). Normality and outliers were analyzed by the Kolmogorov–Smirnov test and Grubb’s method (*α* = 0.05), respectively. Statistical comparisons were performed using the two-tailed *t*-test, the Mann–Whitney *U* test, two-way ANOVA (Tukey post-hoc test). Linear regressions, Spearman and Pearson tests were applied to analyze the correlations. The variables and factors in this study were: age, sex, disease, volume, α-synucleinopathy (α-syn), neurodegeneration (Neu-N), microglial (Iba-1) and astroglial (GFAP) inflammation, disease duration, and post-mortem interval. Data are represented as the mean ± SD, and the differences were regarded as statistically significant at *p < 0.05 and **p < 0.01.

## Supplementary information

Supplementary figures

## Data Availability

The data that support the findings of this study are available from the corresponding author upon reasonable request.
