# Supplementary material for: Astrogliosis and sexually dimorphic neurodegeneration and microgliosis in the olfactory bulb in Parkinson’s disease
Source: NPJ Parkinsons Dis. 2021 Jan 21;7:11. doi: 10.1038/s41531-020-00154-7 (PMC7820595; doi:10.1038/s41531-020-00154-7)
Supplement: Supplementary file 1 — Supplementary figures [file 41531_2020_154_MOESM1_ESM.pdf]

Supplementary figure 1. Correlations of Neu-N, GFAP and Iba-1 compared to α-synuclein

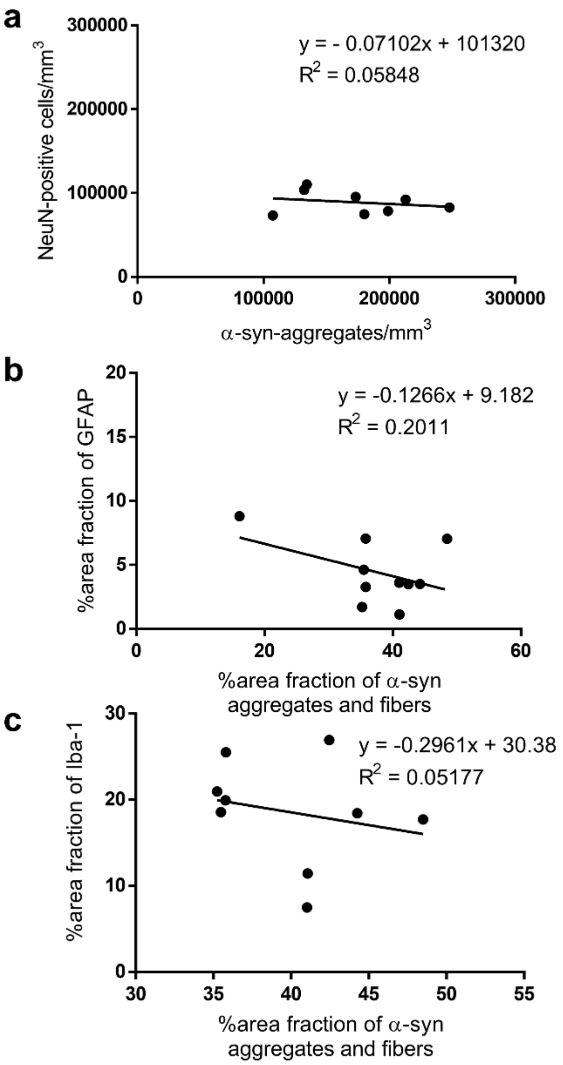

Supplementary Fig. 1. Correlations of Neu-N (cells/mm^3) compared to α-synuclein (aggregates/mm^3) (a), percentage of area fraction of GFAP against percentage of area fraction of α-synuclein (b) and finally, percentage of area fraction of Iba-1 against percentage of area fraction of α-synuclein (c).

Supplementary figure 2. Correlations of α-synuclein, Neu-N, GFAP, Iba-1 compared to disease duration.

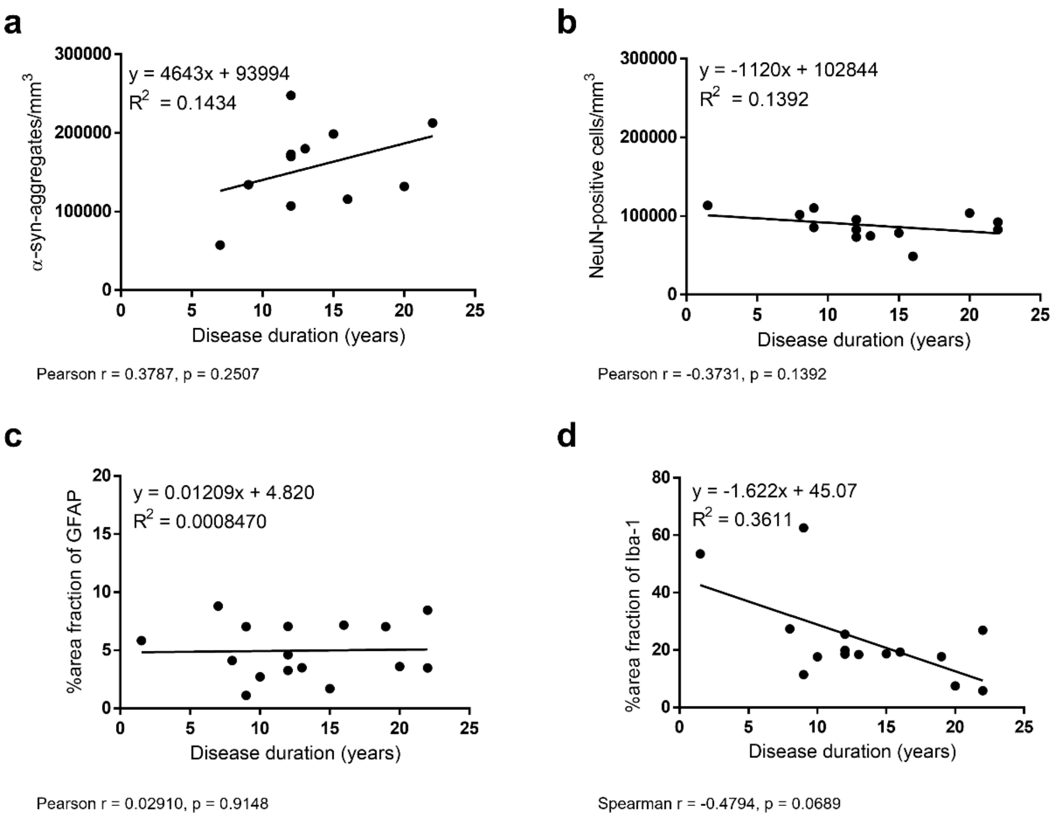

Supplementary Fig. 2. Correlations of α-synuclein (aggregates/mm^3), Neu-N (cells/mm^3), GFAP (percentage of area fraction) and Iba-1 (percentage of area fraction) compared to disease duration, using Pearson and Spearman tests. No substantial correlations were found.

Supplementary figure 3. Correlations of α-synuclein, Neu-N, GFAP, Iba-1 compared to post-mortem interval.

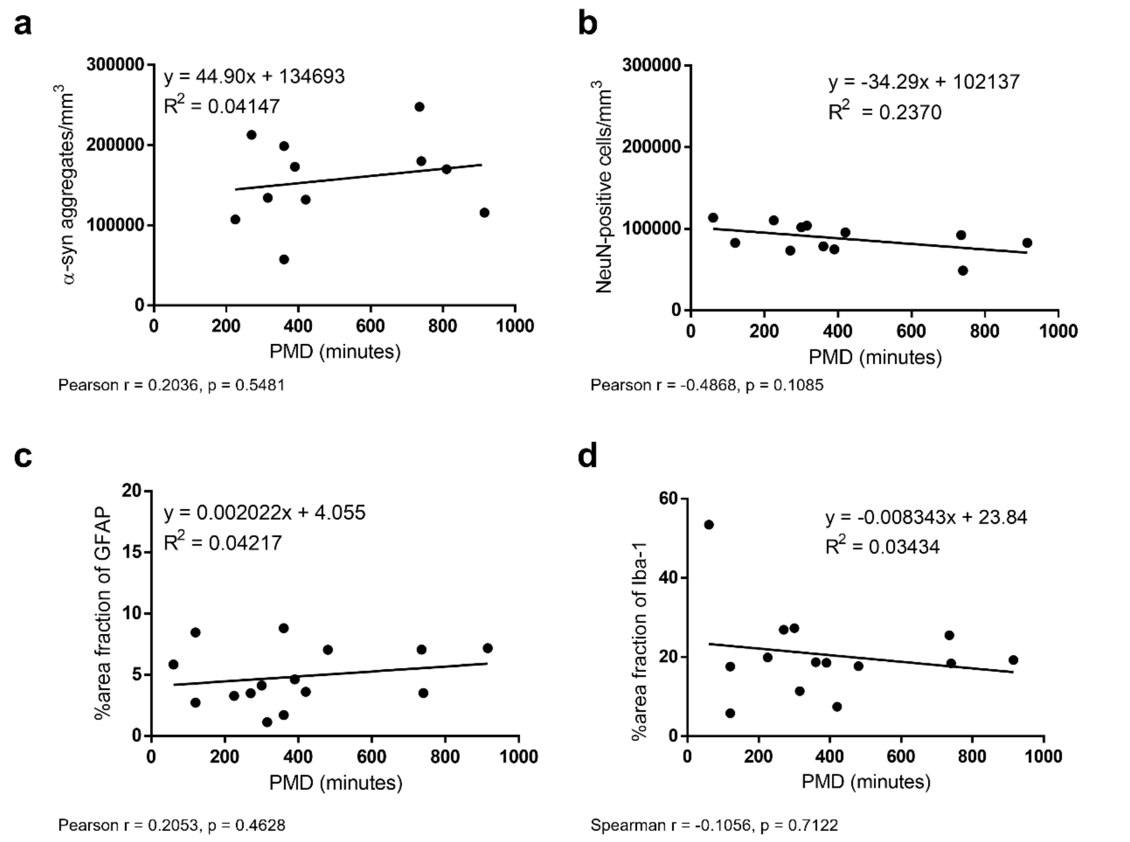

Supplementary Fig. 3. Correlations of α-synuclein (aggregates/mm^3), Neu-N (cells/mm^3), GFAP (percentage of area fraction) and Iba-1 (percentage of area fraction) compared to post-mortem interval, using Pearson and Spearman tests. No correlations were detected.

Supplementary Figure 4. Stereological area fraction fractionator

a

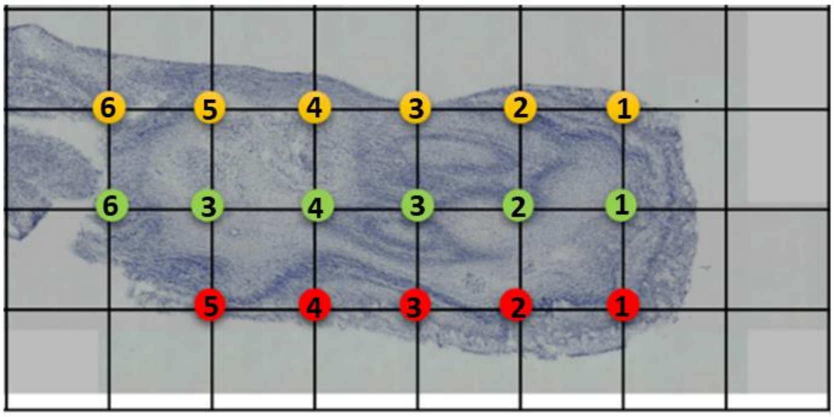

b

Case	Random number Excel f_x=RAND()	Even/odd number
1	0.93	Odd number
2	0.68	Even number
3	0.73	Even number
4	0.77	Odd number
.	.	.
.	.	.
.	.	.

c

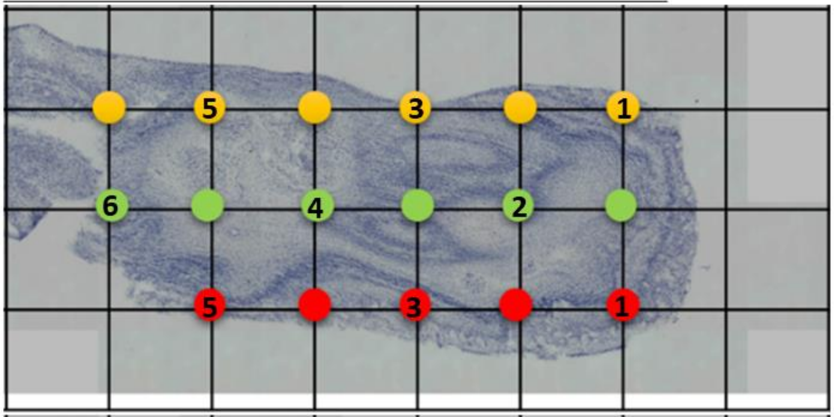

d

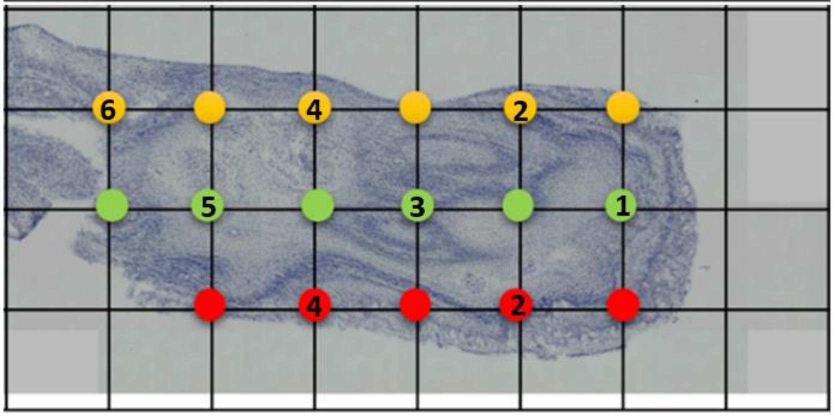

Supplementary Fig. 4. Stereological area fraction fractionator probe to quantify GFAP and Iba-1 staining. Following an unbiased protocol: a millimetric transparent grid simulating an array was randomly overlapped in the slide and crossed matched to the tissue identified (black lines) (a); odd and even numbers were randomly assigned to each human case (b). In odd-numbered cases, the images were taken from the first cross-matching of the tissue following this cross-matching sequence for the first line: 1-3-5-7 (c). In even-numbered cases, the images were captured in the second cross-matching following this sequence: 2-4-6-8 (d). Images were processed with an ImageJ protocol to obtain the percentage of area fraction.
